# FOSL1 is a novel mediator of endotoxin/lipopolysaccharide-induced pulmonary angiogenic signaling

**DOI:** 10.1038/s41598-020-69735-z

**Published:** 2020-08-04

**Authors:** Christopher R. Nitkin, Sheng Xia, Heather Menden, Wei Yu, Min Xiong, Daniel P. Heruth, Shui Qing Ye, Venkatesh Sampath

**Affiliations:** 1 Division of Neonatology, Children’s Mercy Kansas City, 2401 Gillham Road, Kansas City, MO 64108 USA; 2 Division of Experimental and Translational Genetics, Children’s Mercy Kansas City, Kansas City, MO 64108 USA; 3 Present Address: Unaffiliated, Kansas City, USA

**Keywords:** Molecular biology, Cell signalling

## Abstract

Systemic sepsis is a known risk factor for bronchopulmonary dysplasia (BPD) in premature infants, a disease characterized by dysregulated angiogenesis and impaired vascular and alveolar development. We have previoulsy reported that systemic endotoxin dysregulates pulmonary angiogenesis resulting in alveolar simplification mimicking BPD in neonatal mice, but the underlying mechanisms remain unclear. We undertook an unbiased discovery approach to identify novel signaling pathways programming sepsis-induced deviant lung angiogenesis. Pulmonary endothelial cells (EC) were isolated for RNA-Seq from newborn C57BL/6 mice treated with intraperitoneal lipopolysaccharide (LPS) to mimic systemic sepsis. LPS significantly differentially-regulated 269 genes after 6 h, and 1,934 genes after 24 h. Using bioinformatics, we linked 6 h genes previously unknown to be modulated by LPS to 24 h genes known to regulate angiogenesis/vasculogenesis to identify pathways programming deviant angiogenesis. An immortalized primary human lung EC (HPMEC-im) line was generated by SV40 transduction to facilitate mechanistic studies. RT-PCR and transcription factor binding analysis identified FOSL1 (FOS like 1) as a transcriptional regulator of LPS-induced downstream angiogenic or vasculogenic genes. Over-expression and silencing studies of *FOSL1* in immortalized and primary HPMEC demonstrated that baseline and LPS-induced expression of *ADAM8*, *CXCR2*, *HPX*, *LRG1*, *PROK2*, and *RNF213* was regulated by FOSL1. *FOSL1* silencing impaired LPS-induced in vitro HPMEC angiogenesis. In conclusion, we identified FOSL1 as a novel regulator of sepsis-induced deviant angiogenic signaling in mouse lung EC and human fetal HPMEC.

## Introduction

Bronchopulmonary dysplasia (BPD) is a developmental lung disorder characterized by simplified alveoli and dysmorphic pulmonary vasculature^1–6^. BPD affects approximately 40% of infants born at ≤ 28 weeks gestational age with up to 29% mortality^7^. Recent studies have found that maternal chorioamnionitis increases the incidence of BPD and perinatal mortality^8^, and postnatal sepsis or pneumonia increases the risk of preterm infants developing BPD^9^. While oxygen toxicity, mechanical ventilation, and inflammation are traditional risk factors for the development of BPD, systemic sepsis has emerged as a significant risk factor for BPD^10–13^. Systemic sepsis caused by Gram-positive and Gram-negative bacteria is common in premature infants, and is associated with BPD^9,14–16^. in addition to airway colonization with Gram negative bacilli correlates with severe BPD^17^. However, the mechanisms underlying sepsis-induced neonatal acute lung injury and alveolar remodeling seen in BPD remain understudied^3,18–21^. We have previously shown that postnatal systemic lipopolysaccharide (LPS) disrupts lung development in newborn mice, leading to alveolar simplification in a Nox2-dependent manner^1^. However, relatively little is known about the impact of LPS on the developing lung vasculature.

In human BPD and hyperoxia models of experimental BPD, several researchers have reported that angiogenesis is impaired and vessel density is decreased^6,22,23^. However, other studies have implicated that dysangiogenesis with increased microvascular growth but dysmorphic arborization is seen in human disease^24,25^. Our prior work suggests that systemic LPS in mice programs a dysangiogenesis phenotype, which also results in alveolar remodeling^26^.

Inflammation alters vascular development, as infants with BPD who experienced sepsis have increased risk of pulmonary hypertension^27^ and biomarkers of angiogenesis such as VEGFA, KDR, endostatin, and angiopoietin-2 are altered in infants who develop subsequently BPD^28^. Despite the accumulating evidence for sepsis-induced aberrant angiogenesis to program vascular remodeling in the developing lung, the mechanisms by which LPS disrupts angiogenesis or programs dysangiogensis vascular phenotypes in the developing lung reamain unclear. Therefore, the objective of this study was to identify novel pathways by which systemic sepsis programs aberrant angiogenesis in the developing lung using an unbiased approach involving RNA-Seq and bioinformatics methods. By combining discovery-based RNA sequencing studies in a neonatal mouse model of sterile sepsis complemented by validation strategies using genetic manipulation in primary fetal human lung endothelial cell (EC) and an immortalized lung EC line we developed, we identify the transcription factor FOSL1 (FOS like 1, also known as FRA-1, FOS-related antigen 1), as a novel regulator of endotoxin-mediated dysangiogenesis in the developing lung.

## Results

### Lung endothelium transcriptome profiling identifies inflammatory, immune, and several pathways differentially regulated by systemic endotoxin in the developing lung at 6 h

**Figure 1:**
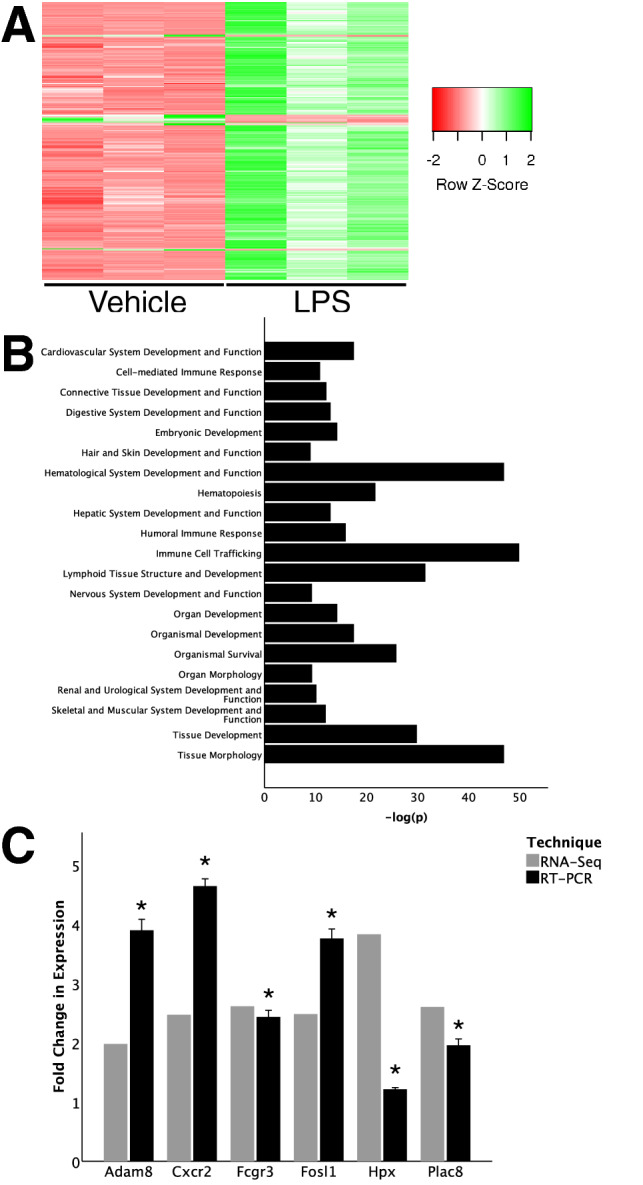
RNA-seq data from lung endothelial cells isolated from DOL-4 mice treated with LPS for 6 h. (**A**) Heatmap of differentially expressed genes (n = 3); (**B**) top pathways identified in Ingenuity Pathway Analysis as activated; and (**C**) validation RT-PCR in primary mouse lung endothelial cells (n = 6). Data presented at mean ± standard deviation, *p < 0.001 between LPS-treated and saline control baseline by one-sample, two-tailed Student’s *t* test as all data were normally distributed. Graphs created in SPSS 26 for Mac (https://www.ibm.com/analytics/spss-statistics-software) and image assembled in Adobe Illustrator 24.1.0 for Mac (https://www.adobe.com/products/illustrator.html).

To identify novel targets induced by systemic endotoxin, mouse lung EC were isolated from 4-day old (DOL-4) LPS-treated (2 mg/kg intraperitoneal, i.p) and control littermates (n = 3/group) after 6 h, and RNA-Seq was performed. Ingenuity Pathway Analysis (IPA) was used to analyze 24,392 genes, of which 269 were significantly up- or down-regulated with *p* and *q* < 0.05 (Fig. 1A). IPA-predicted physiological system development and functional categories induced by LPS were mainly involved in leukocyte recruitment and function, as expected for a pro-inflammatory stimulus (Table S1). Angiogenesis as a component of “Cardiovascular System Development and Function” was confirmed as predicted increased with LPS treatment, significant across the data set at 6 h (p = 2.84 × 10^–18^) with + 3.422 z-score of activation (Fig. 1B).

After preliminary identification of significant changes of gene expression, the IPA Knowledge Base (KB) of ~ 5.9 million citations was used to efficiently filter RNA-Seq gene expression data. We targeted those genes that were previously not known to be altered with LPS treatment and showed significant changes. This yielded 97 genes that were significantly up- and down-regulated with p and q < 0.05 (Table S2). We validated 6/97 targets, identified by RNA-Seq with RT-PCR (Real-Time PCR); *Fosl1*, *Adam8*, *Cxcr2*, *Plac8*, *Fcgr3*, and *Hpx* in primary murine pulmonary EC isolated under similar experimental conditions as RNA-seq data in independent mouse samples (n = 6/group) (Fig. 1C). Genes were chosen for validation based on below described IPA analysis linking 6 h and 24 h sequencing data.

### Lung endothelium transcriptome profiling identifies angiogenic or vasculogenic genes differentially regulated in the neonatal lung with systemic endotoxin at 24 h

**Figure 2:**
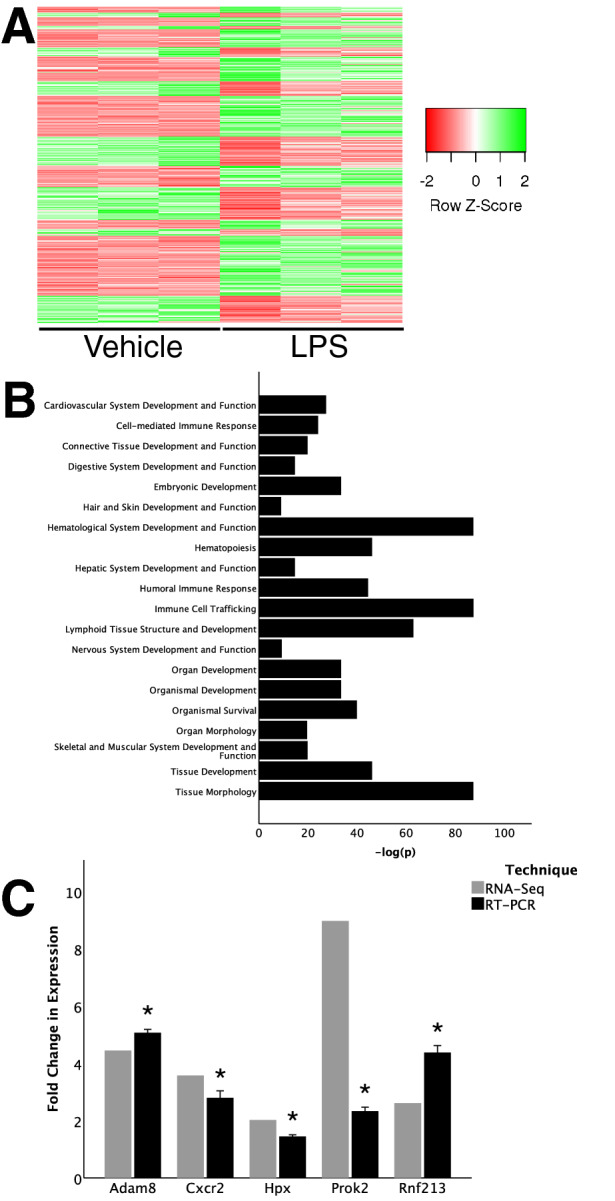
RNA-seq data from lung endothelial cells isolated from DOL-4 mice treated with LPS for 24 h. (**A**) Heatmap of differentially expressed genes (n = 3); (**B**) top pathways identified in Ingenuity Pathway Analysis as activated; and (**C**) validation RT-PCR in primary mouse lung endothelial cells (n = 6). Data presented at mean ± standard deviation, *p < 0.001 between LPS-treated and saline control baseline by one-sample, two-tailed Student’s *t* test as all data were normally distributed). Graphs created in SPSS 26 for Mac (https://www.ibm.com/analytics/spss-statistics-software) and image assembled in Adobe Illustrator 24.1.0 for Mac (https://www.adobe.com/products/illustrator.html).

To identify novel LPS-induced angiogenesis and vasculogenesis pathways, RNA-Seq was performed as above on mouse lung EC isolated from LPS-treated and control DOL-4 mice 24 h after LPS exposure. IPA identified 1,934 genes which were significantly up- or down-regulated with p and q < 0.05 (Fig. 2A). IPA-predicted physiological system development and function categories induced by LPS were mainly involved in leukocyte recruitment and function (Table S3). Angiogenesis as a component of “Cardiovascular System Development and Function” was confirmed as predicted increased with LPS treatment, significant across the data set at 24 h (p = 2.68 × 10^–26^) with + 2.474 z-score of activation (Fig. 2B). As before, IPA was used to efficiently filter RNA-Seq gene expression data, identifying genes known to be important in angiogenesis or vasculogenesis. This yielded 235 genes (see Table S4). Using Pubmed searches, we identified *Adam8*, *Cxcr2*, *Rnf213*, and *Hpx* as genes previously implicated in regulating angiogenesis or vasculogenesis in developmental or pathological conditions, but not reportedly downstream of LPS or its receptor Toll Like receptor 4. We validated expression of these genes in independent mouse lung EC samples obtained 24 h after i.p. LPS injection (Fig. 2C).

### Discovery of novel transcriptional links between LPS and angiogenesis/vasculogenesis

**Figure 3:**
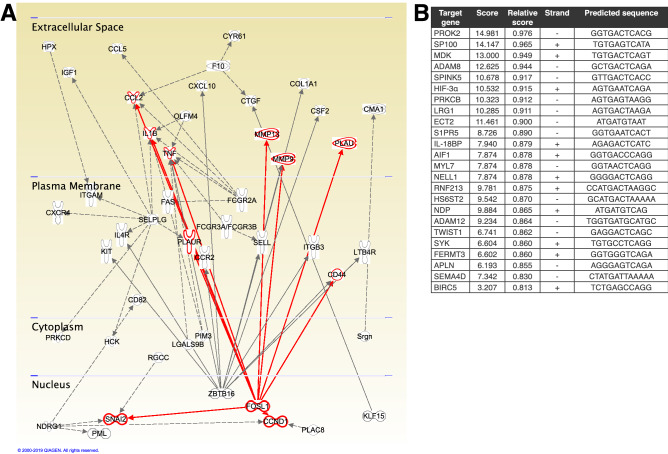
Bioinformatics analysis of data. (**A**) IPA-generated map linking FOSL1 to angiogenesis or vasculogenesis genes; (**B**) Identification of targets downstream of FOSL1 by JASPAR ranked by score. Image created in Ingenuity Pathway Analysis (https://digitalinsights.qiagen.com/products-overview/discovery-insights-portfolio/analysis-and-visualization/qiagen-ipa/) and assembled in Adobe Illustrator 24.1.0 for Mac (https://www.adobe.com/products/illustrator.html).

As we were investigating novel links between genes induced by LPS at 6 h that can transcriptionally upregulate angiogenic/vasculogenic genes at 24 h, we used IPA to make connections between genes at 6 h not known to be induced by LPS, and genes at 24 h known to be involved in angiogenesis or vasculogenesis (Fig. 3A). Only genes found to have ≥ 50% increase in expression were pursued. Connections between 6 and 24 h genes were based solely on transcriptional regulation in the IPA. Non-transcriptional interactions such as protein–protein interactions or phosphorylation, etc. were initially explored but not pursued, and are described in the Supplementary material (Table S5). Additionally, to maximize impact, only 6 h genes with ≥ 3 downstream targets at 24 h were investigated, while genes with < 3 downstream targets are shown in the Supplementary material (Table S5).

**Figure 4:**
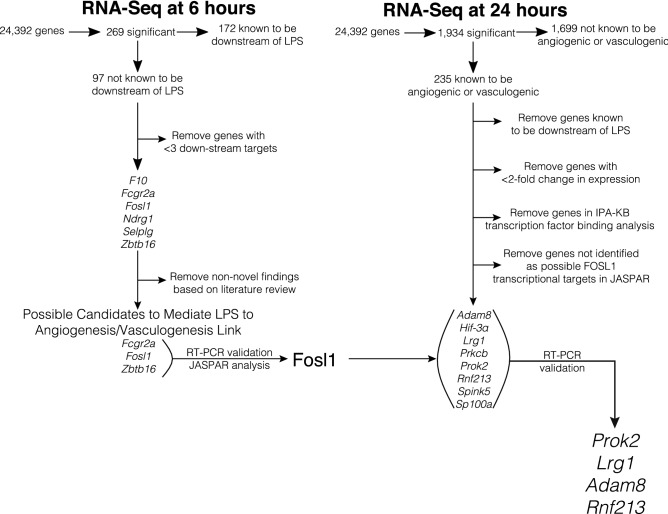
Flowchart of gene identification. Describing identification of genes of interest, starting from identification of genes with statistically significant changes in expression at 6 h that were not known to be influenced by LPS, removal of non-novel findings, RT-PCR validation and linkage to genes with statistically significant changes in expression at 24 h that were known to be angiogenic or vasculogenic, had at least two-fold change in expression, and not found to be linked to *Fosl1* or LPS in the IPA-KB, validated by RT-PCR resulted in the final list of *Prok2, Lrg1, Adam8,* and *Rnf213.* Image created in Adobe Illustrator 24.1.0 for Mac (https://www.adobe.com/products/illustrator.html).

Six genes (*F10*, *Fcgr2a*, *Fosl1*, *Ndrg1*, *Selplg*, *Zbtb16*) were induced 6 h after LPS in mouse lung EC, and found to have ≥ 3 transcriptional relationships with 24 h downstream angiogenic/vasculogenic targets. Three of these targets (*F10*, *Ndrg1*, *Selplg*) were eliminated based on literature review, as these were not novel LPS/TLR targets . This left us with *Fcgr2a*, *Fosl1*, and *Zbtb16* as potential target genes induced early with LPS that transcriptionally regulated 24 h angiogenic genes (Fig. 4). *Fcgr2a* is not a transcription factor, *Zbtb16* was not validated by RT-PCR, so these were excluded. This left *Fosl1* as the sole remaining target.

### Creation of immortalized human pulmonary lung endothelial cell line (HPMEC-im)

**Figure 5:**
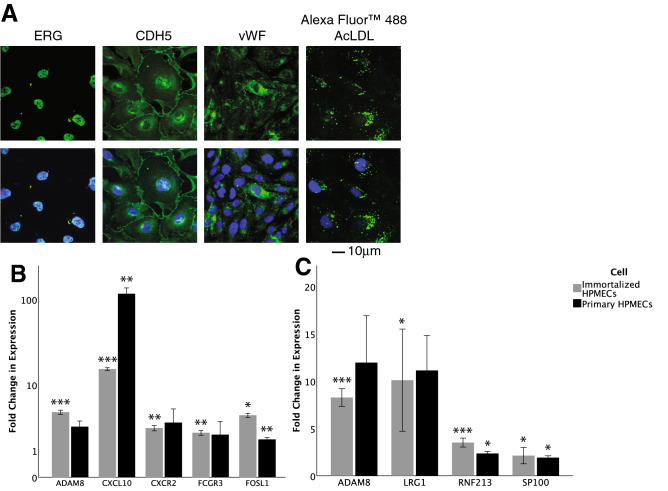
Validation of cell line as endothelial cells. (**A**) expression of ERG, CDH5, and vWF by immunoflourescence, and uptake of AcLDL; (**B**) assessment of novel LPS genes after treatment of primary HPMECs (n = 3) and HPMEC line (n = 4) with LPS for 6 h (p values determined by one-sample, two-tailed Student’s *t* test); and (**C**) assessment of angiogenesis or vasculogenesis genes after treatment of primary HPMECs (n = 3) and HPMEC line (n = 5) with LPS for 24 h (p values determined by one-sample, two-tailed Student’s *t* test; all data were normally distributed except 24 h primary HPMEC LRG1 which was assessed with one-sample Wilcoxon signed rank test). Data presented at mean ± standard deviation. *p < 0.05, **p < 0.01, ***p < 0.001 between LPS-treated and saline control baseline. Graphs created in SPSS 26 for Mac (https://www.ibm.com/analytics/spss-statistics-software) and image assembled in Adobe Illustrator 24.1.0 for Mac (https://www.adobe.com/products/illustrator.html).

We proceeded to establish functional relationships between FOSL1 and downstream targets in an in vitro model. We used 19-week gestation, male primary HPMEC (ScienCell), derived from neonatal lungs. However, these cells do not allow plasmid-mediated genetic manipulation studies as they are fragile. We therefore used primary HPMEC to generate HPMEC-im using SV40 large antigen transformation. Ets-related gene (*ERG*) and *VCAM-1* expression, and oxidized lipoprotein uptake confirmed preservation of EC phenotype (Fig. 5A). To extend RNA-Seq and IPA findings into human cells and to serve as a baseline for mechanistic studies, RT-PCR assessment of both the 6 h genes not previously known to be regulated by LPS and the 24 h known angiogenesis and vasculogenesis genes was undertaken.

### RT-PCR assessment of novel genes activated by LPS linked to angiogenesis and vasculogenesis genes in HPMEC-im

Five genes identified from our 6 h RNA sequencing data were assessed with RT-PCR in HPMECs, including *FOSL1*, *ADAM8*, and *FCGR3*, as well as *CXCR2* and *CXCL10* which were included for validating LPS-induced cytokine expression (Fig. 5B). *FOSL1* had multiple multiple connections to differentially-expressed 24 h angiogenesis genes, and it was the only transcription factor identified. Therefore, *FOSL1* (FOS like 1, Fra-1, AP-1 transcription factor subunit) was further explored as an early EC gene upregulated with LPS that could potentially induce expression of downstream genes involved in angiogenesis and vasculogenesis. IPA-KB analysis of FOSL1 and the 235 angiogenesis/vasculogenesis genes at 24 h above revealed ten of the 24 h genes had a transcriptional relationship with FOSL1: *Ccl2, Ccnd1, Cd44, Il-1β, Mmp13, Mmp9, Plau, Plaur, Snai2,* and *TNF.* However, many of these have been reported to be linked with LPS in the literature (e.g., *Mmp-9*), did not seem relevant to angiogenesis (e.g., *Cd44*), or were not specific for angiogenesis (e.g., *Tnf*, *Il-1β*). Therefore, the list of 24 h angiogenic/ vasculogenic genes was expanded beyond those only predicted by IPA to be linked to 6 h genes.

We then focused our search to genes (a) not reported to be downstream of LPS, (b) with at least a two-fold increase in expression, and (c) not known to be linked to FOSL1 in the IPA-KB. This resulted in a list of 15 potential genes of interest: *Adam8, Aif1, Fermt3, Hif-3α, Il-18bp, Lrg1, Myl7, Prkcb, Prok2, Rnf213, S1pr5, Sema4d, Spink5, Syk,* and *Sp100a*. As these novel relationships were outside of the validated IPA-KB, JASPAR analysis was performed to identify transcriptional relationships from *FOSL1*, and indicated eight of these genes may have FOSL1-binding sites in the human promoter (Fig. 3B), including *ADAM8, HIF-3α, LRG1, PRKCB, PROK2, SPINK5, AND SP100A* (Fig. 4). *RNF213* and *HPX* were also included based on previous preliminary Kyoto Encyclopedia of Genes and Genomes (KEGG) analysis. Of these, four angiogenesis/vasculogenesis genes (*ADAM8*, *MDK*, *RNF213*, *SP100*) were confirmed to be upregulated with LPS in primary HPMEC (18 weeks gestation, female) and HPMEC-im (19-week gestation, male) (Fig. 5C) by RT-PCR.

### O***verexpression of FOSL1 increases expression of angiogenesis and vasculogenesis genes***

**Figure 6:**
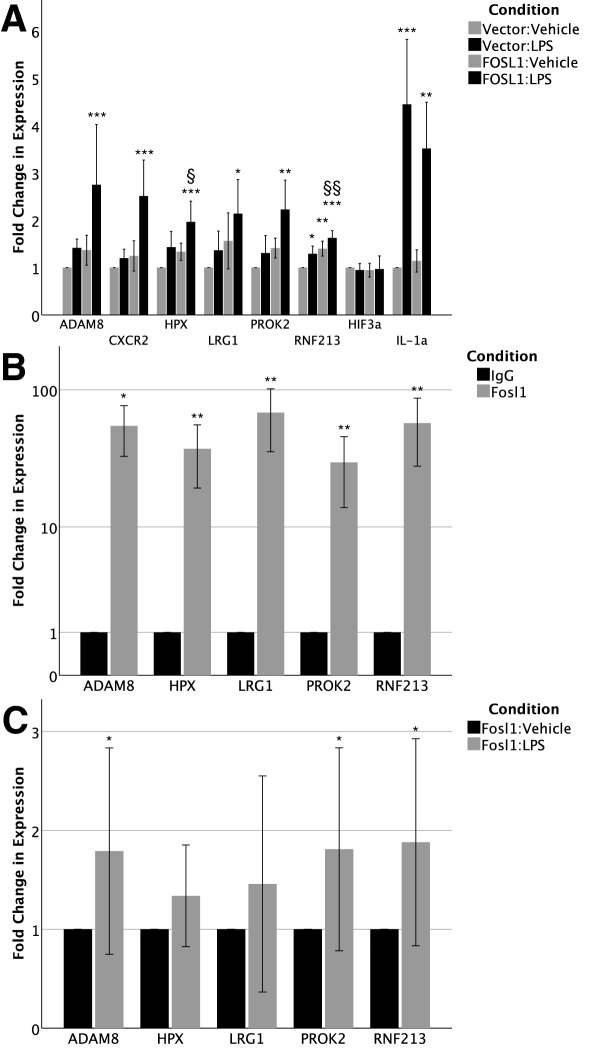
FOSL1 overexpression results. Overexpression of FOSL1 in HPMEC-im experiments, including (**A**) effect of LPS treatment, FOSL1 overexpression, and combined LPS treatment plus FOSL1 overexpression on angiogenesis or vasculogenesis gene expression (n = 5 per condition) (*p < 0.05 between LPS-treated and saline control; ^§^p < 0.05, ^§§^p < 0.01 between vector:LPS and FOSL1:LPS; by one-way ANOVA and post-hoc Tukey tests [HPX, RNF213, IL-1α] or Krukal-Wallis and post-hoc Mann–Whitney tests [ADAM8, CXCR2, LRG1, PROK2]); (**B**) ChIP experiments demonstrating pull-down of downstream target genes by FOSL1 (n = 5 per condition, presented as mean ± standard deviation with *p < 0.05, **p < 0.01, ***p < 0.001 by one-sample, two-tailed Student’s *t* test [LRG1, PROK2, RNF213] or one-sample Wilcoxon signed rank test [ADAM8]; and (**C**) ChIP experiments demonstrating increased binding of FOSL1 to target gene promoters after LPS stimulation (n = 5 per condition, presented as mean ± standard deviation with *p < 0.05, **p < 0.01, ***p < 0.001 by one-sample Wilcoxon signed rank test). Graphs created in SPSS 26 for Mac (https://www.ibm.com/analytics/spss-statistics-software) and image assembled in Adobe Illustrator 24.1.0 for Mac (https://www.adobe.com/products/illustrator.html).

To validate our JASPAR analysis and investigate whether *FOSL1* actually regulates the in silico*-*identified downstream targets, we used an overexpression strategy. An expression plasmid containing *FOSL1* cDNA was cloned and transfected in HPMEC-im with and without LPS treatment. We obtained > 150 -fold increased expression of *FOSL1* with transfection in HPMEC-im [C + vector vs. C + *Fosl1*; 1.05 ± 0.2 vs. 159 ± 55.3, < 0.001, n = 5]. Similarly, we noted ≈ 200 fold increase in *FOSL1* expression in LPS-treated HPMEC-Im [LPS + vector vs. LPs + *Fosl1*; 2.065 ± 0.56 vs. 199 ± 70.4, < 0.001, n = 5]. RT-PCR of angiogenesis or vasculogenesis genes revealed that *FOSL1* overexpression alone stimulated increased gene expression of *ADAM8*, *CXCR2*, *HPX*, *LRG1*, *PROK2*, and *RNF213* (Fig. 6A) by 24–56% above non-transfected controls, though not significantly. Compared to *FOSL1* overexpression or LPS treatment independently, combined *FOSL1* overexpression and LPS treatment resulted in significant induction of *ADAM8*, *CXCR2*, *HPX*, *LRG1*, *PROK2*, and *RNF213* in HPMEC-im (Fig. 6A). Finally, combined *FOSL1* overexpression and LPS treatment resulted in significant increases in gene expression compared to controls without *FOSL1* transfection or LPS treatment. *HIF-3α* expression did not change with FOSL1 overexpression, and served as negative control. *IL-1α* expression, which is known to be negatively regulated by FOSL1^29^, served as an additional control to validate FOSL1-dependent LPS-mediated gene expression in HPMEC. These data demonstrate that *FOSL1* transcriptionally regulates native, and synergistically, LPS-sensitive gene expression of identified targets.

To investigate whether FOSL1 directly binds to the promoter region of identified targets after LPS treatment we performed chromatin immunoprecipitation (ChIP). We noted that FOSL1 robustly bound to the promoters of *ADAM8, HPX, LRG1, PROK2,* and *RNF213* at baseline, indicating baseline regulation of these targets by FOSL1 (Fig. 6B). FOSL1 binding to the promoter regions of *ADAM8*, *PROK2*, and *RNF213* increased by 86.7 to 100.4%, respectively, 7 h after LPS treatment in HPMEC-im, suggesting LPS-induced transcriptional activation of genes in HPMEC-im (Fig. 6C). Increased expression of downstream angiogenic genes with FOSL1 overexpression, and binding of FOSL1 to the promoter regions of target genes support the role of FOSL1 in mediating LPS-induced lung EC angiogenenic signaling.

### FOSL1 silencing decreases LPS-induced expression of angiogenesis and vasculogenesis genes

**Figure 7:**
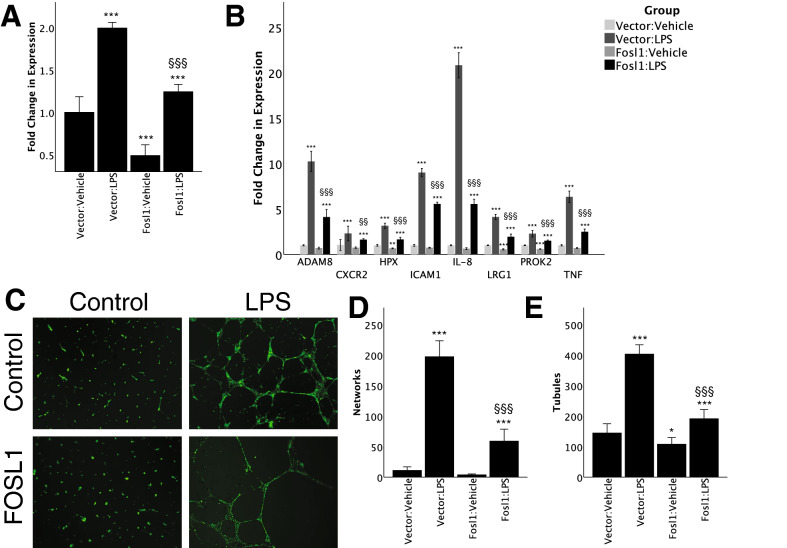
FOSL1 silencing results. siRNA silencing of FOSL1 experiments, including (**A**) demonstration of FOSL1 knock-down (n = 4, by one-way ANOVA and post-hoc Tukey tests); (**B**) effect of LPS treatment, FOSL1 overexpression, and combined LPS treatment plus FOSL1 overexpression on angiogenesis or vasculogenesis gene expression (n = 4 per condition, by one-way ANOVA and post-hoc Tukey tests); and representative (**C**) immunofluorescence images and quantification of (**D**) network (n = 4 per condition; by one-way ANOVA and post-hoc Tukey tests) and (**E**) tubule formation of angiogenesis forming assays. *p < 0.05, **p < 0.01, ***p < 0.001 between LPS-treated and saline control; ^§^p < 0.05, ^§§^p < 0.01, ^§§§^p < 0.001 between vector:LPS and FOSL1:LPS. Data presented at mean ± standard deviation. Graphs created in SPSS 26 for Mac (https://www.ibm.com/analytics/spss-statistics-software) and image assembled in Adobe Illustrator 24.1.0 for Mac (https://www.adobe.com/products/illustrator.html).

We next explored a loss of function approach to complement our overexpression studies. Here, primary HPMECs (not transformed) were transfected with *FOSL1*-siRNA with and without LPS treatment. After confirming knockdown [C vs. si-FOSL1; 1.0 ± 0.1 vs. 0.49 ± 0.06, p < 0.03, n = 4] of *FOSL1* expression (Fig. 7A), RT-PCR of angiogenesis or vasculogenesis genes was carried out. Silencing *FOSL1* alone significantly reduced expression of *ADAM8*, *HPX*, *LRG1*, *CXCR2*, and *PROK2* by 26–41% compared to scrambled siRNA controls. *FOSL1* silencing suppressed LPS-induced expression of *ADAM8*, *HPX*, *LRG1*, *CXCR2*, and *PROK2* (Fig. 7B) by 29–73%. *IL8*, *TNFα*, and *ICAM-1* were included for validation as positive controls. These data confirm the LPS-induced expression of angiogenic and vasculogenic genes is suppressed by silencing *FOSL1* in primary lung EC. Taken in conjunction with prior overexpression data in HPMEC-im, these data imply that induction of target genes after LPS treatment is *FOSL1*-dependent.

We next examined the impact of *FOSL1* in regulating angiogenic sprouting in primary HPMEC by pursuing a matrigel-based angiogenesis assay in HPMEC. LPS induced robust in vitro angiogenesis in matrigel as evidenced by increased branches and networks (confluence of ≥ 2 branches). LPS-induced angiogenesis was strongly suppressed by *FOSL1* silencing (Fig. 7C–E). These data demonstrate that *FOSL1* regulates LPS-induced in vitro angiogenesis.

## Discussion

Although sepsis is known to promote the vascular and alveolar remodeling underlying bronchopulmonary dysplasia, the mechanisms by which inflammation can modulate pulmonary angiogenesis have not been fully defined. In this unbiased, hypothesis-generating study, we performed transcriptome profiling on lung EC in a rodent model of systemic sepsis, and identified FOSL1 as a novel early target gene not previously described to be transcriptionally regulated by LPS. This novel transcriptional regulator was then linked to the angiogenesis or vasculogenesis genes *LRG1*, *RNF213*, and *Sp100*, which have previously not been reported to be downstream of LPS or TLR signaling. Complementary overexpression and silencing approaches in a HPMEC-im line we generated as well as primary fetal HPMEC validated the LPS-FOSL1-angiogenesis/vasculogenesis pathway by revealing dependence of basal angiogenesis and vasculogenesis gene expression on *FOSL1*. Finally, impaired in vitro angiogenesis in *FOSL1*-deficient ECs strongly implicates the transcription factor *FOSL1* as a central mediator of inflammation-induced pulmonary EC angiogenesis.

Previous studies in premature infants and experimental models of BPD suggest that dysregulation of angiogenesis is important for BPD pathogenesis^3,18^. For example, in rodent models of BPD, hyperoxia leads to decreased angiogenesis through ERK1/2-dependent signaling^19^. Similarly, hyperoxia variably induces expression of angiogenic genes such as the angiopoietins, platelet derived growth factors, and vascular endothelial growth factor in rats^20^, culminating in a BPD-like phenotype. Angiopoietin-1 has been shown to ameliorate experimental BPD in a rodent model^21^, while angiopoietin-2 worsens hyperoxia-mediated lung injury in nitric oxide- synthase 2-deficient mice^30^, and increased angiopoietin-2 is associated with BPD in preterm infants^31^. Our group has previously shown that LPS treatment of newborn rodents recapitulates the alveolar simplification and impaired vascularization as observed in clinical BPD, by stimulating aberrant angiogenesis via TLR4-ERK-FOXC2-DLL4 signaling^26,32^. Others have found LPS treatment of newborn sheep to decrease lung *CTGF* expression and result in abnormal lung development^33^ similar to BPD. While studies suggest inflammatory angiogenesis contributes to vascular and alveolar remodeling in the developing lung, transcriptional regulation of sepsis-induced deviant lung angiogenic networks implicated in pathological vascular remodeling has not been previously investigated. In this study, we identified FOSL1 as a novel regulator of the pulmonary angiogenic response.

FOSL1 is one of several subunits that binds a Jun-family member to form the AP-1 complex. FOSL1 has important effects in osteoclasts and disorders of bone via Wnt signaling^34–36^ and mediates anti-inflammatory actions of some natural compounds^37–39^. *FOSL1* is also a proto-oncogene, affecting the ability of cancer cells to invade and metastasize in sarcomas^40^, squamous cells carcinomas^41^, melanoma^42^, gastric cancer^43^, pancreatic cancer^44^, and lung cancer^44^. With respect to the endothelium, it is known that *Fosl1*^−/−^ embryos die and develop vascular defects in extraembryonic tissues^45^. Accordingly, embryonic stem cells lacking *FOSL1* differentiate into ECs but do not form primitive capillaries or tube-like structures and *FOSL1* is a required for HUVEC assembly into vessels^45^. FOSL1 has also been reported as modulating angiogenesis-vasculogenesis and β3 integrin and endothelial cell adhesion^46^, but this study is the first report of FOSL1 acting as a transcriptional regulator of angiogenesis-related genes. Additionally, we extend existing work^46^ by including knock-down and over-expression experiments. While an interaction between LPS and *FOSL1* in modulating gefitinib-induced interstitial lung disease has been reported^47^, whether LPS directly regulates FOSL1-dependent signaling and the role of FOSL1 in mediating LPS-induced expression of key downstream angiogenic targets has not been previously investigated.

We identified *PROK2*, *RNF213*, and *LRG1* as novel targets which downstream of LPS, transcriptionally regulated by FOSL1, and are implicated in angiogenesis/vasculogenesis. *PROK2*^48^ is an important contributor to tumor angiogenesis. Relevant to neonatal disease, *LRG1*^49^ may contribute to the aberrant retinal vasculature observed in oxygen-induced retinopathy^49^, while *RNF213*^50^ is required for normal vascular development. Of these, *LRG1* is the only overlapping reported finding in the literature where *FOSL1* knockdown in HUVECs decreased *LRG1* to the same degree that we found in our immortalized HPMEC line. The current study places this understanding within the context of neonatal sepsis and implicates *FOSL1* not only in embryonic development, but also potentially as a mediator of impaired vascular development in the neonatal lung. We further explored this relationship between LPS, FOSL1, and angiogenesis/vasculogenesis with primary HPMEC and an immortalized HPMEC line we derived from primary HPMEC. Not only did *FOSL1* overexpression increase sensitivity to LPS stimulation, *FOSL1* overexpression alone induced angiogenic or vasculogenic gene expression, indicating basal regulation. Additionally, using a ChIP assay, we demonstrated specific binding of FOSL1 to promoters of *PROK2*, *ADAM8*, *RNF213*, *HPX,* and *LRG1* downstream targets at baseline, and increased binding to *PROK2*, *ADAM8*, *RNF213* promoters after LPS treatment. That all genes did not demonstrate additional binding with LPS treatment could represent differences in time kinetics of binding after LPS treatment. Differences in the degree of LPS-induced target gene expression between mice and HPMEC could result from the influence of other lung cell types and cytokines in vivo, timing of i.p LPS in vivo vs. direct LPS in vitro, and encapture of several RNA transcripts by RNA sequencing as against qRT-PCR. Similary, differences between primary HPMEC and HPMEC-Im may have arisen because of faster proliferation in HPMEC-im and the potential for multiple gene copies in HPMEC-im. However, the direction and amplitude of gene expression changes with LPS were not hugely different considering these variables.

A prior study in adult mice with constitutive over-expression of *FOSL1* resulted in rapid death after high-dose LPS treatment (50 mg/kg) with massive inflammation and chemokine response^47^. However, this study did not report any findings related to expression of angiogenesis genes or angiogenic phenotype^47^. In previous studies from our lab, a 25-fold lower dose of i.p. LPS, as in this study, did not result in significant lethality, but a more nuanced alteration of the angiogenic/vasculogenic phenotype with decreased lung EC population^51^. The dosing we used is more consistent with the clinical scenario in preterm infants, where most infants survive Gram-negative sepsis. Herein, we propose aberrant pulmonary angiogenesis as a possible mechanism contributing to this response. Conversely, mice deficient in *Fosl1* are less susceptible to LPS-induced lung injury and mortality^52^, with lower lung injury responses, fewer neutrophils, less NFκB and cJun-AP1 binding^52^, and lower *IL-1β* and *MIP-1α* expression and higher *IL-10* expression^53^. Our in vivo results in HPMEC is consistent with these study in showing that pro-inflammatory genes induced with LPS are suppressed or increased after *FOSL1* silencing or overexpression, respectively. Our data support enhanced inflammation-induced angiogenesis/vasculogenesis as an important mechanism of altered *FOSL1* expression. Manipulation of *FOSL1* expression may therefore lead to modulation of response to inflammation. The lack of readily-available *Fosl1*^+*/−*^ mice (Fosl1^−/−^ mice have embryonic lethality) and inducible EC-specific conditional *Fosl1* knockout mice precluded us from performing experiments to confirm our in vitro findings. Validation of our in vitro findings in mice with *Fosl1* deficiency in vivo would have strengthened our conclusions. Work by Schreiber et al. demonstrating a role for *Fosl1* in placental vascular development^54^ is however, consistent with our results.

This study has several strengths. Use of unbiased bioinformatics tools to generate hypotheses allowed us to quickly identify novel targets, which could then be explored with standard laboratory techniques in both murine and human cells, including a newly created and validated HPMEC line. Additionally, demonstration of direct binding of FOSL1 to promoter genes, and loss- and gain-of-function approachs to examine relationships between LPS, FOSL1 and target angiogenesis/vasculogenesis pathway genes confirmed regulation. Finally, validation of *FOSL1* as a mediator of sepsis-induced aberrant angiogenesis in human EC enhances clinical relevance. A limitation is our initial reliance on the IPA knowledge base, which is periodically, not continuously, updated; however, confirmation of IPA findings with more up-to-date literature searches was undertaken. Examining how FOSL1 gets activated by LPS, investigating protein level changes of target genes, and their individual roles in LPS-induced angiogenesis are interesting avenues for future research. Finally, while embryonic lethality of *Fosl1*^*−/−*^ mice precluded in vivo mechanistic validation of our results, studies in mice with conditionally-deleted endothelial *Fosl1* would strengthen our data.

In conclusion, we have identified *FOSL1* as a novel, early, central target in inflammation-induced angiogenesis in primary lung EC and HPMECs, linking sepsis with expression of multiple genes involved with aberrant angiogenesis in the developing lung, including *ADAM8*, *LRG1*, *RNF213*, *HPX*, *CXCR2*, *PROK2*, and *SP100*, which may contribute to development of BPD. Taken together, these data indicate a strong, central role of *FOSL1* in regulating lung EC angiogenesis in the context of inflammation. While hyperoxia and sepsis are associated with vascular remodeling and BPD, they may alter lung angiogenesis potentially differently. Hyperoxia disrupts angiogenesis, while sepsis may program a dysangiogenesis phenotype, wherein there might be increased but non-directional dysmorphic angiogenesis^22–25^. Our prior work and the results of this study that systemic LPS may program dysangiogenesis lung phenotypes that may also impair normal vascular development. The role of FOSL1 in mediating sepsis-induced pulmonary dysangiogenesis and vascular remodeling using EC-specific conditional FOSL1 knock down mice as well as the role of FOSL1 in regulating inflammatory angiogenesis in other organs are topics or future research. Our data support testing whether short-term inhibition of EC-FOSLI can serve as a therapeutic target to prevent development of vascular defects in BPD.

## Methods

### Cell culture and reagents

Human primary pulmonary microvascular endothelial cells (HPMEC) were purchased from a commercial vendor (ScienCell, Carlsbad, CA) and used as previously described^32^. These HPMECs were derived from lungs of newborn infants, and only cells between passages 3 and 4 were used for all experiments. Primary HPMEC used for our experiments were derived from the lungs of 18 weeks female fetus. HPMECs were grown in endothelial cell medium supplemented with 5% fetal bovine serum (FBS), antibiotics, and endothelial cell growth serum as recommended by the manufacturer in a humidified incubator containing 5% CO_2_ at 37 °C. Ultrapure lipopolysaccharide (LPS, 100 ng/mL) was purchased commercially from Invivogen (San Diego, CA).

### Animal model of sepsis-induced lung injury in neonatal mice

Wild-type C57BL/6 mice were obtained commercially from Charles River (Burlington, MA). For all animal experiments, half the litter of pups were injected with LPS (2 mg/kg) and other half injected with sterile saline (controls) intra-peritoneally (i.p.) (Sigma, St Louis, MO) served as littermate controls, as previously performed^51^. We did not observe any mortality in our experiments. The pups were left with the dam and closely monitored for signs of distress for half a hour, and then they were observed briefly once more on the same day for the 24 h experiments. All animal experiments were performed on mouse pups on DOL 4. Mice were sacrificed using a i.p. injection of pentobarbital (100 mg/kg) and were exsanguinated after cessation of heartbeat. The lungs were harvested for EC isolation.

### Isolation of murine endothelial cells

Each endothelial preparaton was from pooled lungs of two C57BL/6 pups (4 days old). 3–4 biological replicates, 6–8 mice/condition was used for experiments. The protocol for the isolation of mouse lung EC was described previously^55^. Briefly, the lungs from euthanized pups were minced with scissors in DMEM and transferred to pre-warmed 1 mg/mL collagenase solution and rotated at 37 °C for 45 min^55^. The digested mixture was passed through a 14 g cannula several times, strained through a 70 µm cell strainer, and washed with a 20% FBS solution^55^. The supernatant was then centrifuged at 400 × *g* for 5 min and the pellet was resuspended with 0.1% bovine serum albumin^55^. The endothelial cells were isolated with anti-PECAM-1 antibody-conjugated Dynabeads (Thermo-Fisher, Rockford, IL) on a rocker for 15 min at room temperature as per the manufacturer’s protocol. RNA was extracted following standard protocol as described below.

### Generation of HPMEC cell line

Primary HPMEC (male, 19 week fetus) were immortalized using SV40 large T antigen. Lentiviral transduction (Applied Biological Materials (ABM), Richmond, BC, Canada). These endothelial cells were cultured in endothelial cell media as above and only cells less than passage ten were used. To validate immortalized HPMEC (HPMEC-im), we cultured cells on coverslips and stained them with antibodies of endothelial markers, such as CDH5, ERG and VWF. The slides were used for IF staining using the primary antibodies (Abcam, Cambridge, MA) with the corresponding Alexa Fluor secondary antibodies (Thermo-Fisher). The slides mounted in Prolong Gold with DAPI (Thermo-Fisher), which stains the nucleus. We also verified IM HMPEC with Alexa Fluor 488 AcLDL uptake experiment. Images were taken at 63× magnification using a Zeiss LSM510 confocal microscope with an attached camera.

### RNA isolation and cDNA generation

Total RNA was extracted from HPMECs and mouse lung EC or tissue using the PureLink RNA Mini Kit (Thermo-Fisher) following the manufacturer’s instructions as previously described^32^. cDNA was synthesized from 1 μg of RNA using a SuperScript IV First-Strand Synthesis Kit (Thermo-Fisher) per the manufacturer’s recommendations.

### RNA-Seq

Total RNA was extracted from mouse lung EC using the mirVana miRNA isolation kit (Thermo-Fisher). RNA sequencing was performed on a HiSeq 1,500 system (Illumina, San Diego, CA). Mapping of RNA-Seq reads and transcript assembly and abundance estimation were conducted using the Tuxedo Suite software package (Broad Institute, Cambridge, MA) as previously described^56–58^. DAVID was used for biological function analysis. Heatmaps were generated from heatmapper.ca/expression and are scaled by row and hierarchically clustered by average linkage distance via Euclidean measurement. Full sequencing data (BioProject ID PRJNA600007) are available online at https://www.ncbi.nlm.nih.gov/bioproject/600007.

### Ingenuity pathway analysis

Networks were generated through the use of IPA (QIAGEN Inc., https://www.qiagenbioinformatics.com/products/ingenuity-pathway-analysis)^59^.

### RT-PCR

Primary mouse pulmonary microvascular EC were used to validate RNA-Seq data. Immortalized and primary human pulmonary microvascular EC were used for mechanistic studies. RT-PCR was performed on cDNA using PowerUp SYBR Green master mix (Thermo-Fisher) and appropriate primers with the SYBR green method on a Thermo Fisher QuantStudio 3. Mouse primers for *Fosl1*, *Adam8*, *Cxcr2*, *Plac8*, *Fcgr3*, *Hpx*, *Rnf213*, and *Prok2*, and human primers for *CXCL10*, *CXCR2*, *ADAM8*, *FOSL1*, *FCGR3*, *FCGR2*, *EFHD2*, *LRG1*, *HIF3A*, *MDK*, *IL1A*, *SP100*, *PROK2*, *HPX*, *IL8*, *ICAM1*, and *TNF* were pre-validated and purchased commercially from Sigma. *Actin* or *18S* were used as housekeeping genes. Relative gene expression was calculated using the Pflaffl method^60^.

### JASPAR analysis

The promotor sequences (about 2 kB region upstream of transcription start site) were downloaded from NCBI genome browser. FOSL1 binding sites on the promoters were predicted by JASPAR 2018^61^ with a threshold of 0.9.

### FOSL1 plasmid generation and transfection

Human *FOSL1* cDNA was amplified and cloned into pIRES-EGFP-Puro (Addgene, Cambridge, MA) with 5′-ACTGCTAGCCACCATGACCTCAACCGGCCAGGATTCCA-3′ and 5′-TGAGAGCTCTTAGTGTGGGTGGGGCATATCCTCCCCAAA-3′ as performed previously^51^. HPMEC grown in 6-well tissue culture plates were transfected overnight with 2 µg of the indicated plasmids or empty plasmids (mock) with Lipofectamine 3,000 (Thermo-Fisher) as per the manufacturer’s protocol. Cells were allowed to recover for 24 h, and were then treated with LPS for 6 or 24 h. Cell lysates were used for RNA quantification by RT-PCR.

### Chromatin immunoprecipitation assay

The ChIP assay was performed as previously described^26^. Briefly, the HPMEC cells were fixed with 1% formaldehyde for 10 min after 7 h LPS treatment at 500 ng/mL. The Pierce Magnetic ChIP Kit wa used according to the manufacturer’s instructions. The FOSL1 antibody (PCRP-FOSL1-1E3) was obtained from Hybridoma Bank. The ChIP products were analyzed by quantitative real-time PCR. The sequences of primers used in RT-PCR are shown below: *ADAM8*, sense-TCCCAGGATAACGTCCGAG and antisense-GAGTCAGGGAACTGCACG; *HPX*, sense-CATCTGTGAGGGATCAGGG and antisense-TGTGAGATTTGCCTAGTGAGTC; *LRG1*, sense-AGGTGTTCATGACAGAGCTG and antisense-CCAATAGTGAGTAATGCCAAACG; *PROK2*, sense-AGCAAGTTCGGTGTGGTC and antisense-AAGTGACAGATTTGCCCTCC; *RNF213*, sense-TGCCCAACTAGCGTTCTAAAG and antisense-GTCTCAAACTCCTGACCTCAG.

### siRNA-mediated FOSL1 gene silencing

siRNA sequences targeting human *FOSL1* (si*FOSL1*) and control si*RNA* were purchased from Santa Cruz Biotechnology (SCBT) (Dallas, TX) and gene silencing was performed following manufacturers recommendations as reported before^32,62^. For the non-silenced cells, control siRNA (SCBT) was used according to the manufacturer’s protocol^54,55^. Briefly, HPMECs were cultured until 80% confluent and the cells were incubated with 8 μg of either control siRNA or si*FOSL1* in transfection medium for 16 h and was changed to normal ECM^54,55^. The cells were grown for another 48 h and treated with LPS for the experiments. The efficiency of the silencing was determined by qPCR.

### Angiogenic tube and network formation assay on Matrigel

The lung EC in vitro angiogenesis network formation assay using a 2 dimensional matrigel platfrom was performed as described previously^32,63^. Briefly, HPMEC were grown to ~ 80% confluence, and then subsequently silenced with si*FOSL1* or control siRNA^32,63^. Cells were treated with LPS for 10 h. Subsequently, cells were detached with TrypLE Express (Thermo-Fisher), re-suspended in basal ECM, and 6 × 10^4^ cells were plated onto a 24-well Matrigel-coated plate (Corning, Corning, MA)^32,63^. After 12 h, angiogenesis was assessed with the use of calcein AM fluorescent dye (Corning), per the manufactuer’s protocol, to enhance the visibility of tube and network formation. Angiogenic quantification was evaluated by counting the number of tube and network formations in one quadrant (the same one for each condition) and multiplying by four^32,63^. For measurements, tubes were considered to be tubular structures connecting two cell clusters, and networks were counted by cell clusters with at least three tubular structures emanating out. Representative images were taken using an Olympus 1 × 71 fluorescence microscope with attached camera at 4× zoom.

### Statistical analysis

All experiments were performed with ≥ 3 biological replicates, as indicated in figure legends, and ≥ 2 technical replicates for RT-PCR. Data were analyzed with SPSS version 26 for Mac (IBM). The Shapiro–Wilk test was used to determine normality. Comparisons between two groups were made by one-sample, two-tailed Student’s *t* test or one-sample Wilcoxon signed rank test for parametric or non-parametric data, respectively. Comparisons between three or more groups, as for the *FOSL1* overexpression and silencing studies, were analyzed by one-way ANOVA and post-hoc Tukey tests for multiple comparisons. All values are expressed as mean ± 1 standard deviation. *P* values < 0.05 were considered statistically significant.

### Ethical approval

Care of mice before and during experimental procedures was conducted in accordance with the policies at the University of Missouri-Kansas City Lab Animal Resource Center and the National Institutes of Health *Guidelines for the Care and Use of Laboratory Animals*. Protocols had prior approval from the University of Missouri-Kansas City Institutional Animal Care and Use Committee.

## Supplementary information

Supplementary Table S1.

Supplementary Table S2.

Supplementary Table S3.

Supplementary Table S4.

Supplementary Table S5.

## Data Availability

The datasets generated during and analysed during the current study are available in the BioProject repository, https://www.ncbi.nlm.nih.gov/bioproject/600007.
